# Effects of Sputtering Parameters on AlN Film Growth on Flexible Hastelloy Tapes by Two-Step Deposition Technique

**DOI:** 10.3390/ma9080686

**Published:** 2016-08-10

**Authors:** Bin Peng, Dongdong Gong, Wanli Zhang, Jianying Jiang, Lin Shu, Yahui Zhang

**Affiliations:** State Key Laboratory of Electronic Thin Films and Integrated Devices, University of Electronic Science and Technology of China, Chengdu 610054, China; gong90dong12@gmail.com (D.G.); wlzhang@uestc.edu.cn (W.Z.); jiangjianying19921@gmail.com (J.J.); s89s89s@gmail.com (L.S.); yahuizhang1990@gmail.com (Y.Z.)

**Keywords:** aluminum nitride thin films, c-axis orientation, Hastelloy tapes, two-step deposition technique, growth mechanism

## Abstract

AlN thin films were deposited on flexible Hastelloy tapes and Si (100) substrate by middle-frequency magnetron sputtering. A layer of Y_2_O_3_ films was used as a buffer layer for the Hastelloy tapes. A two-step deposition technique was used to prepare the AlN films. The effects of deposition parameters such as sputtering power, N_2_/Ar flow rate and sputtering pressure on the microstructure of the AlN thin films were systematically investigated. The results show that the dependency of the full width at half maximum (FWHM) of AlN/Y_2_O_3_/Hastelloy on the sputtering parameters is similar to that of AlN/Si (100). The FWHM of the AlN (002) peak of the prepared AlN films decreases with increasing sputtering power. The FWHM decreases with the increase of the N_2_/Ar flow rate or sputtering pressure, and increases with the further increase of the N_2_/Ar flow rate or sputtering pressure. The FWHM of the AlN/Y_2_O_3_/Hastelloy prepared under optimized parameters is only 3.7° and its root mean square (RMS) roughness is 5.46 nm. Based on the experimental results, the growth mechanism of AlN thin films prepared by the two-step deposition process was explored. This work would assist us in understanding the AlN film’s growth mechanism of the two-step deposition process, preparing highly c-axis–oriented AlN films on flexible metal tapes and developing flexible surface acoustic wave (SAW) sensors from an application perspective.

## 1. Introduction

Recently, with the rapid development of science and technology, sensors for harsh environment applications are greatly required to sense physical quantities such as temperature, strain, torque, etc., in the metallurgy [1], petrochemical [2] and aerospace industries [3]. For example, in combustion chambers and turbine systems, as well as in rocket engines, sensors that can work at high temperatures and in harsh environments are really needed. Due to their high sensitivity [4] and wireless [5] and passive operation [6], surface acoustic wave (SAW) sensors can work in these harsh environments, especially for rotating components. To measure complex or curved surfaces, flexible film SAW sensors are preferred. Aluminum nitride (AlN) has a high SAW velocity, breakdown dielectric strength, etc., which makes it promising for the application of thin films SAW sensors [7]. So far, lots of studies on flexible film SAW sensors have been reported [8,9]. However, all these flexible SAW sensors are deposited on organic substrates which would fail to work at high temperatures. So we propose to fabricate SAW sensors with AlN thin films which are deposited on a flexible alloy substrate such as Hastelloy tapes. As a result, it can meet the demand of high temperature and flexibility. Lots of studies have been done to deposit AlN thin films on nonmetallic substrates such as silicon [10], sapphire [11] and polymer substrates [12]. However, due to the big difference between the properties of ceramics and those of metal substrates, such as lattice mismatches and thermal expansion coefficients, it is hard to prepare AlN ceramic films of high quality on metal substrates.

In our previous work, we proposed a two-step deposition process to deposit AlN films on a metal substrate [13]. In addition, a Y_2_O_3_ buffer layer was used to prepare AlN thin films on a rough metal substrate [14]. However, its growth mechanism on metal substrates is not clear. In this work, the effects of sputtering parameters on the microstructure of AlN films are systematically studied and the AlN thin film’s growth mechanism of the two-step deposition process is explored. Such a work may help us understand the growth mechanism of AlN films on an alloy substrate, thereby paving an new way to integrate function films and electric devices with metal components or workpieces.

## 2. Materials and Methods

**Figure 1:**
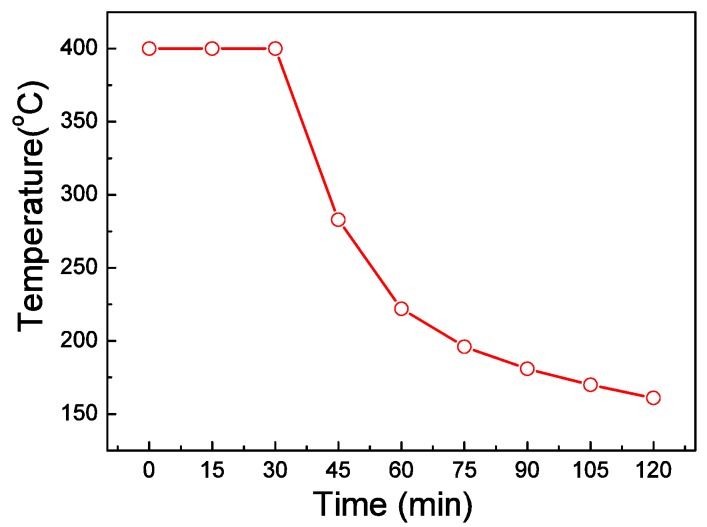
Dependence of substrate temperature on sputtering time.

AlN thin films were deposited by middle-frequency (40 kHz) magnetron sputtering. The substrates used in this work were Hastelloy C-276 alloy tapes, which consist of 16.0% of molybdenum, 15.8% of chromium, 5.3% of iron, 3.8% of wolfram and 59.1% of nickel. The size of the Hastelloy substrates is 40 mm × 10 mm and its thickness is about 0.1 mm. The Hastelloy alloy tapes were polished by electrolytic process firstly. Then the Hastelloy tapes were planarized by Y_2_O_3_ buffer layer using solution deposition method before deposition of AlN films. The details of the solution deposition method can be found in reference [15]. The role of the Y_2_O_3_ layer is to provide flat surface for the subsequent deposition of AlN films. The effects of the Y_2_O_3_ buffer layer on the quality of the AlN films had been reported in our earlier work [14]. The sputtering target is Aluminum disk with purity of 99.999%. Before depositing, the sputtering chamber was evacuated to 8 × 10^−4^ Pa. Then the Al target was pre-sputtered for 20 min to clean the surface. High purity N_2_ (99.999%) and Ar (99.999%) were injected into the chamber. A so-called two-step deposition technique [13] was used. Firstly, the AlN films were deposited for 30 min and the substrates temperature was fixed at 400 °C. Secondly, we shut down the substrate heater and AlN films were deposited under natural cooling condition. The substrate temperature was measured with a thermocouple attached underneath the substrate holder during depositing process. The substrate temperature as a function of deposition time was shown in Figure 1. The total sputtering time was 2 h and the thickness of the AlN films was about 3 μm. This two-step deposition process was proposed in reference [13] in detail. Si (100) substrates were also used to deposit AlN films in every sputtering process for comparison.

We characterized the crystal structure of the AlN thin films by X-ray diffraction (XRD) (Cu-Ka, Bede-D1). The full width at half maximum (FWHM) of the AlN (002) diffraction peak was used to characterize the degree of c-axis oriented AlN films perpendicular to the substrates. Atomic force microscopy (AFM) (SPA-300V, Seiko Instrument, Tokyo, Japan) was used to measure the morphologies and RMS surface roughness of the AlN thin films. Scanning electron microscopy (SEM) (Inspect F50, FEI, Shanghai, China) was used to analyze the surface and cross-section microstructures of the AlN thin films.

## 3. Results and Discussion

### 3.1. Sputtering Power

**Figure 2:**
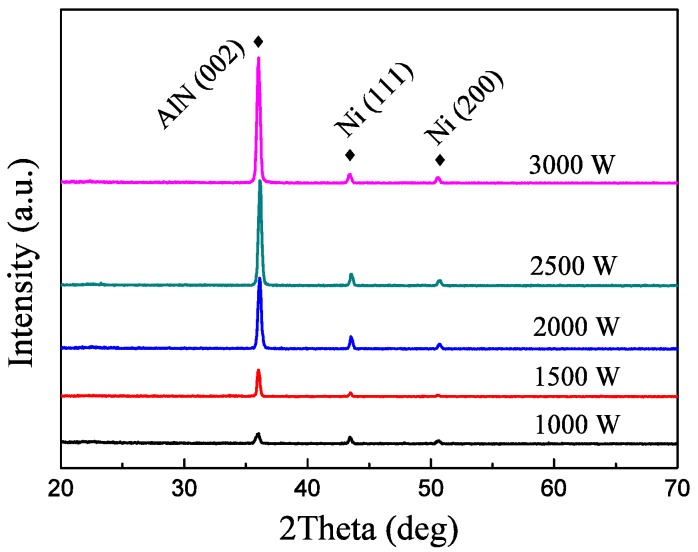
XRD spectra of the AlN films deposited under different sputtering powers.

Figure 2 shows the XRD spectra of the AlN film deposited on Y_2_O_3_/Hastelloy under a deposition pressure of 1.0 Pa, a N_2_/Ar flow ratio of 30:70 Sccm and different sputtering powers. As shown in the XRD pattern, we can only observe the very narrow AlN (002) diffraction peak, as well as Ni (111) and Ni (200). The intensity of the Ni (111) and Ni (200) diffraction peaks is far weaker than that of the AlN (002) peak. We can conclude that all the AlN thin films show c-axis-preferred orientation. It can also be found that the intensity of the AlN (002) diffraction peak increases rapidly with the increase of sputtering power. The intensity of the AlN (002) diffraction peak reaches its maximum when the sputtering power is 3000 W.

**Figure 3:**
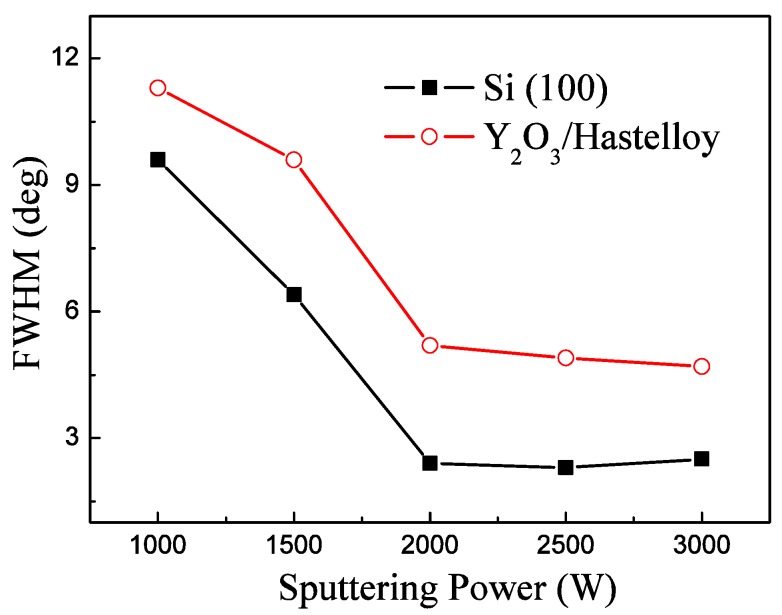
FWHM of the AlN (002) peak of the AlN films as a function of sputtering power.

Figure 3 shows the FWHM of the AlN (002) peak of the AlN films deposited under different sputtering powers. The FWHM is a very important parameter to evaluate the quality of AlN piezoelectric films. The smaller the FWHM, the better the c-axis orientation of the AlN films, thus indicating better piezoelectric properties. We can find that the FWHM of the AlN (002) peak of the AlN films deposited on the Hastelloy tapes falls quickly when the sputtering power increases from 1000 to 2000 W. When the sputtering power is greater than 2000 W, the FWHM varies slowly with sputtering power, which means that the sputtering power has little influence on the FWHM of the c-axis–oriented AlN films in this case. We can observe that the dependence of the FWHM of AlN/Si (100) on the sputtering power is similar to that of AlN/Y_2_O_3_/Hastelloy.

From Figure 2 and Figure 3, we can find that the sputtering power has a profound impact on the quality of AlN films. We think the reason is that the sputtering power mainly affects the primary energy of the sputtered particles arriving at the substrate surface in magnetron sputtering. It is well known that in wurtzite AlN crystalline cells, the formation energy of the Al–N bond in the c-axis direction is greater than that of the other Al–N bond in the (100) direction. Therefore, as Tanner [16] and Iriarte [17] pointed out, the sputtered atoms do not have enough energy to form a (002)-oriented structure if the primary energy is low. However, the sputtered atoms have high energy under high sputtering power and it is easy to form a Al–N bond in the c-axis direction or the (002) orientation [18]. Thus, the grain of the AlN films would grow with a preferred c-axis orientation. 

### 3.2. N_2_/Ar Flow Ratio

**Figure 4:**
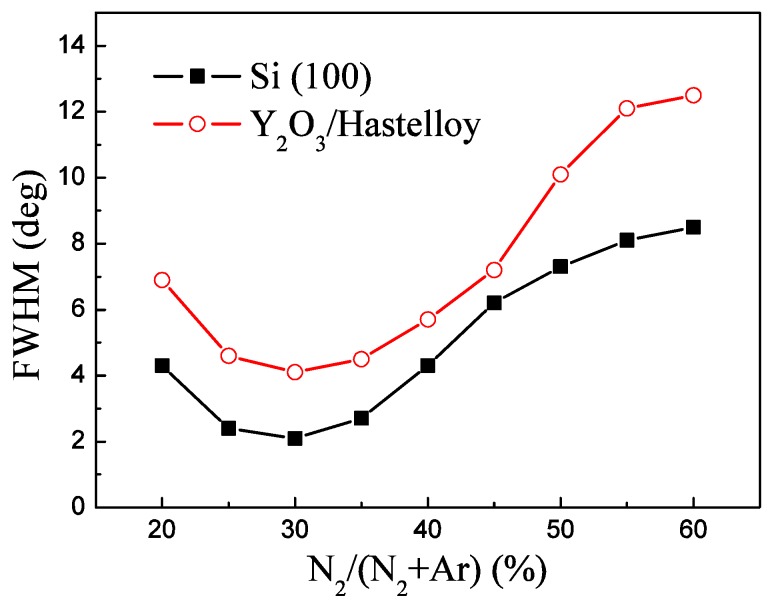
FWHM of the AlN (002) peak of AlN films deposited under different N_2_/Ar flow ratios.

Figure 4 shows the FWHM of the AlN (002) peak of the AlN films deposited under a deposition pressure of 1.0 Pa, a sputtering power of 2000 W and different N_2_/Ar flow ratios. We can find that the FWHM of the AlN (002) peak of AlN/Y_2_O_3_/Hastelloy falls firstly with the increase of the N_2_/Ar flow ratio and then rises with the further increase of the N_2_/Ar flow ratio. The FWHM of the AlN (002) peak reaches its minimum value of 4.1° when the N_2_/(N_2_ + Ar) is 30%. We can find that the dependence of the FWHM of AlN/Si (100) on the N_2_/Ar flow ratio is also similar to that of AlN/Y_2_O_3_/Hastelloy. 

For ideal sputtering, if all the sputtered Al element combines with the N element and forms AlN, the Al/N ratio would be just 1:1 in AlN films. Then it is very important to control the N_2_/Ar flow ratio [19,20]. The influences of the N_2_/Ar flow ratio on the quality of AlN films can be divided into three categories: insufficiency, excess, appropriateness. When the N element is insufficient, the Al element in AlN thin films would be superfluous. On the other hand, too much N_2_ leads to superfluous N elements and Al vacancies in the films. In addition, at high N_2_ concentration conditions, the transfer of energy between sputtering ions and the target atom is low due to the lower mass of N^2+^ than that of Ar^+^. As a result, the sputtered Al particles do not have enough kinetic energy to rearrange atoms in the (002) plane [21]. Therefore, both of these conditions would result in a high density of defects and poor quality of AlN films. Only when the N_2_ concentration is appropriate can highly c-axis–oriented AlN thin films be prepared.

### 3.3. Sputtering Pressure

**Figure 5:**
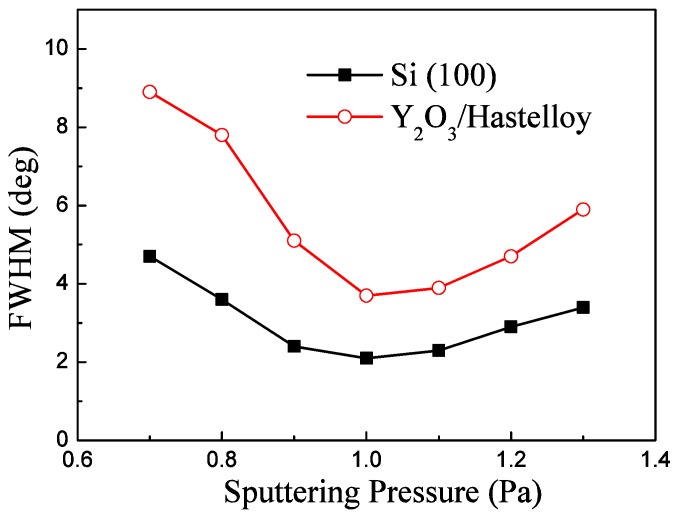
Effects of sputtering pressure on the FWHM of the AlN (002) peak.

Figure 5 shows the FWHM of the AlN (002) peak under a deposition pressure of 1.0 Pa, a N_2_/Ar flow ratio of 30:70 Sccm and different sputtering pressures. As shown in Figure 5, the FWHM of the AlN (002) peak of AlN/Y_2_O_3_/Hastelloy decreases firstly with the increase of sputtering pressure and increases with the further increase of the sputtering pressure. The FWHM reaches its minimum value of 3.7° at 1.0 Pa. It can be seen that the dependence of the FWHM of AlN/Si (100) on the sputtering pressure is also similar to that of AlN/Y_2_O_3_/Hastelloy. 

Sputtering pressure mainly affects the mean free path of sputtered particles. Under high sputtering pressure, the mean free path of sputtered particles becomes short because it is easy to collide with gas molecules, resulting in energy decreasing, while under low sputtering pressure, the mean free path of sputtered particles is long. Thus, the sputtered particles would retain enough energy and this effect can promote the growth of AlN films with a (002) orientation [21]. However, if the sputtering pressure is very low, higher energy sputtered particles due to fewer collisions arrive at the substrate and introduce defects and residual stress in the AlN films [21], which would result in a high FWHM. So there is an optimum sputtering pressure to prepare highly c-axis–oriented AlN films.

### 3.4. AlN Thin Film Growth under Optimized Sputtering Parameters

**Figure 6:**
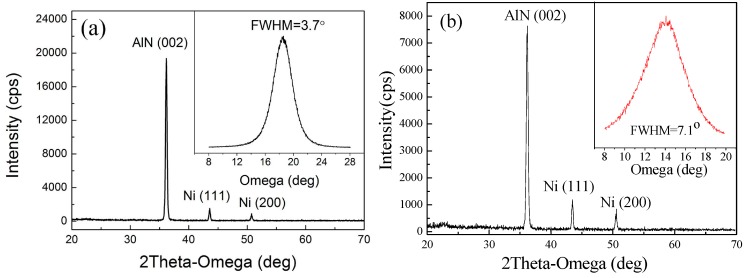
XRD pattern of AlN films deposited on Y_2_O_3_/Hastelloy tapes with (**a**) optimum two-step sputtering parameters and (**b**) single-step growth method at room temperature under the same sputtering parameters.

Based on the above works, highly c-axis–oriented AlN thin films have been deposited on flexible Y_2_O_3_/Hastelloy tapes with the optimized sputtering parameters. The final optimum sputtering parameters were obtained with a sputtering power of 2000 W, a N_2_/Ar flow ratio of 30:70 Sccm and a sputtering pressure of 1.0 Pa. Figure 6a shows the XRD pattern of the AlN thin film deposited with optimized sputtering parameters. The FWHM of the AlN (002) peak of the AlN thin film is only 3.7°, which is good enough to be used to fabricate SAW devices. In comparison with the traditional single-step growth method, AlN films are also deposited at room temperature with a single deposition process and the sputtering parameters are the same as that of the optimum two-step growth method. The XRD pattern of AlN films deposited with the single-step growth method is shown in Figure 6b. We can find that the FWHM of AlN films deposited with the two-step growth method is smaller than that of the single-step growth method. These results indicate that the two-step growth method can enhance the AlN (002) orientation growth, compared with the single-step growth method. 

**Figure 7:**
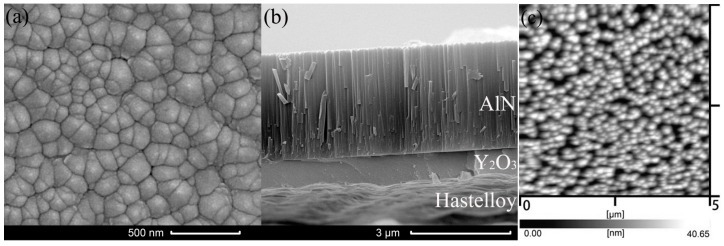
(**a**) SEM surface; (**b**) cross-section SEM micrographs; and (**c**) AFM image of AlN thin films deposited on Y_2_O_3_/Hastelloy tapes with optimum sputtering parameters.

Figure 7a shows the SEM surface morphology of the AlN thin films. We can find that the grain size of AlN films is quite small and homogeneous. Also, we can find that the grains are neatly arranged and compact. Figure 7b shows the cross-section SEM micrographs of the AlN thin film. The AlN film, Y_2_O_3_ buffer layer and Hastelloy substrate can be seen clearly. The thickness of the Y_2_O_3_ buffer layer and the AlN thin films is about 1 µm and 3 µm, respectively. It is obvious that the surface of the Hastelloy tape is very rough, while the interface between the Y_2_O_3_ layer and the AlN thin film is very smooth. In addition, we can see that AlN films exhibit a strong columnar microstructure which is perpendicular to the Y_2_O_3_ buffer layer. Figure 7c shows the AFM image of the AlN films. It is obtained from the AFM image that the grain size is about 247 nm. The surface of the AlN thin films is quite smooth and its RMS roughness is only 5.45 nm.

**Figure 8:**
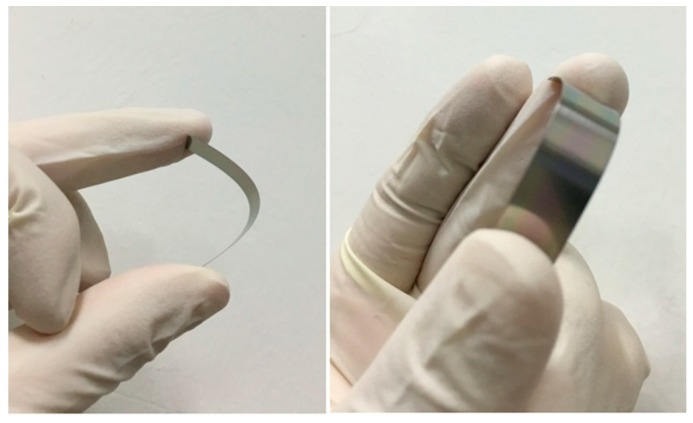
Photos of AlN films deposited on flexible Y_2_O_3_/Hastelloy tapes.

Figure 8 shows the photos of the AlN thin films deposited on flexible Y_2_O_3_/Hastelloy tapes. We can find that the sample can be greatly bent. There is no crack and the AlN films do not delaminate from the Hastelloy tapes when the sample recovers its shape, which suggests that the prepared AlN films can be used to fabricate flexible SAW devices.

### 3.5. Growth Mechanism of AlN Films on Y_2_O_3_/Hastelloy Tapes by Two-Step Process

**Figure 9:**
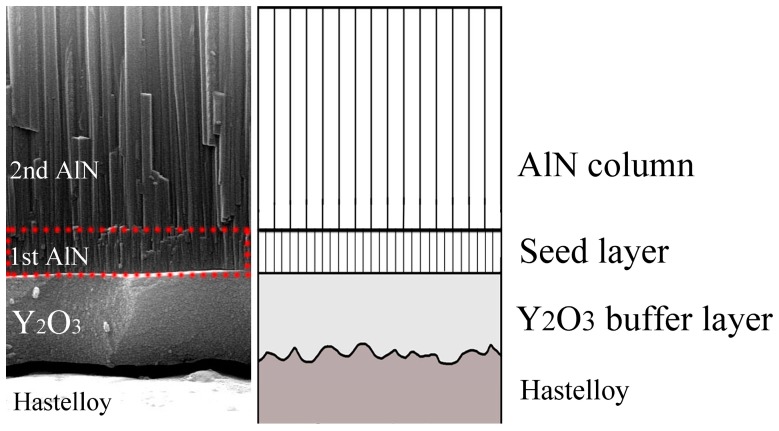
Growth model of AlN thin films on Y_2_O_3_/Hastelloy tapes.

In this section, we will analyze the growth mechanism of c-axis–oriented AlN thin films on from a micro-perspective with our proposed two-step process. Figure 9 shows the cross-section SEM image and the growth model of the AlN thin films. From the cross-section SEM image, we can observe that there are three layers deposited on the Hastelloy tapes. The first layer is the Y_2_O_3_ buffer layer which is deposited on the bare Hastelloy tapes. This amorphous Y_2_O_3_ buffer layer provides a nanoscale flat surface (RMS < 1 nm) for the subsequent deposition of AlN thin films.

In the initial AlN sputtering stage, which corresponds to the first step in our two-step deposition process, the AlN atoms condense with the others to form thin films which have discrete islands with various orientations. Then the islands grow and coalesce with each other to form a thin film having all orientations. The orientation is now dependent on the growth rate. The AlN (002) orientation is preferred because the growth rate of AlN films is high at high temperatures (400 °C). This layer of the AlN film can be clearly seen in the cross-section SEM image, marked as “1st AlN”, and we can regard this layer as the seed layer. This seed layer is like a nucleation layer which can wet the substrate surface, provide a flat surface, produce the nucleation sites and act as a buffer layer to accommodate the lattice mismatch between the substrate and AlN films and promote AlN c-axis–preferred growth [22]. From Figure 3, Figure 4 and Figure 5 we find that the change of the FWHM of AlN/Y_2_O_3_/Hastelloy with the sputtering parameters is similar to that of AlN/Si (100). Now it can be interpreted that this AlN seed layer provides a similar surface for the subsequent growth of AlN films on both the Hastelloy substrate and Si substrate. The results also suggest that highly c-axis-oriented AlN films can be prepared on other substrates with the proposed two-step process. In the second step of our two-step process, the AlN crystalline column grows continuously on the seed layer, and finally forms homogeneous and compactly arranged AlN films with c-axis–preferred orientation, which is the third layer as shown in Figure 9, marked as “2nd AlN”.

## 4. Conclusions 

In this study, we systematically investigated the influence of deposition parameters on the microstructure of AlN thin films and explored the growth mechanism of AlN thin films on Y_2_O_3_/Hastelloy tapes. The results show that the sputtering power has a great influence on the primary energy of the sputtered particles arriving at the substrate surface which is the key energy to form c-axis–oriented AlN columns. Much more or less of the N element would result in poor quality AlN films. The sputtering pressure affects the mean free path of the sputtered particles, and therefore the c-axis orientation. High quality AlN films with c-axis–preferred orientation have been successfully deposited on the Y_2_O_3_/Hastelloy substrate under optimized sputtering parameters. The FWHM of the AlN films deposited under optimized sputtering conditions is only 3.7° and the surface RMS roughness is 5.46 nm. It is found that the dependence of the FWHM of AlN/Y_2_O_3_/Hastelloy on the sputtering parameters is similar to that of AlN/Si (100). The results suggest that highly c-axis–oriented AlN films can also be prepared on other substrates with the proposed two-step process. This work would support us in depositing AlN piezoelectric films on flexible metal tapes and fabricating flexible micro-electro-mechanical system (MEMS)/SAW devices integrated with metal components.
